# The effects of practical vascular blood flow restriction training on skeletal muscle hypertrophy

**DOI:** 10.1186/1550-2783-11-S1-P18

**Published:** 2014-12-01

**Authors:** John O'Halloran, Bill Campbell, Nicholas Martinez, Shane O’Connor, Jonathan Fuentes, N Theilen, J Wilson, M Kilpatrick

**Affiliations:** 1 University of South Florida, Tampa, Florida, USA

## Background

Practical blood flow restriction training is a novel training method that has the potential to increase muscular hypertrophy and muscular strength while allowing individuals to train with lighter loads (20-30% of 1-RM). Through the use of elastic knee wraps, the limbs can be restricted using a perceived pressure scale. The comparison of practical blood flow resistance training with traditional, non-occluded resistance training and its effects on muscle hypertrophy has yet to be investigated. The purpose of this study was to compare the effects of practical vascular blood flow restriction training vs. traditional resistance training (non-blood flow restricted) on skeletal muscle cross sectional area during a 4-week period in trained, college-age males.

## Methods

Twenty-one resistance-trained males volunteered to participate in a 4-week training program and were randomly assigned to one of two groups: Practical vascular blood flow restriction training (BFR; n = 10) and Resistance training (RT; n = 11). The primary difference between the groups was the BFR group performed approximately 60% of all sets blood flow restricted at 20-30% of 1-RM while the RT group performed all sets at an intensity of > 70% 1-RM in a traditional manner (non-blood flow restricted). Perceived pressure for blood flow restriction in the BFR group for the arms and legs was 7 out of 10. Workouts for both groups were similar and consisted of whole body routines. Over the 4-week study period, each participant conducted a total of 11 workouts. Biceps and vastus lateralis muscle cross sectional area was measured via a portable ultrasound device (Bodymetrix Pro, IntelaMetrix). A 2x2 repeated measures ANOVA was used to assess group, time, and group by time interactions for muscle cross-sectional area of the biceps brachaii and the vastus lateralis. Statistical significance was set to p ≤ 0.05. Consent to publish the results was obtained from all participants.

## Results

No significant differences were observed between groups in biceps muscle cross-sectional area (BFR-Pre: 33.2 ± 3.6 mm, BFR-Post: 34.5 ± 4.5 mm, RT-Pre: 31.9 ± 3.3 mm, RT-Post: 33.5 ± 3.7 mm, p = 0.779). However, a significant main effect for time in relation to skeletal muscle hypertrophy for the biceps muscle cross-sectional area was observed (p = 0.004). Similarly, no significant differences were observed between groups in vastus lateralis muscle cross-sectional area hypertrophy (BFR-Pre: 38.1 ± 9.3 mm, BFR-Post: 37.5 ± 9.0 mm, RT-Pre: 36.5 ± 6.8 mm, RT-Post: 35.3 ± 6.1 mm; p = 0.721).

## Conclusion

There were no differences observed between the two training groups in measures of skeletal muscle hypertrophy. However, it is likely that the length of the training program (4 weeks in duration encompassing 11 workouts) was not long enough for differences to emerge in the training techniques, if a difference exists. Future investigations into this area of comparing practical vascular blood flow restriction training to traditional resistance training should utilize a longer period of training.

